# In-Vitro and In-Silico Evaluations of Heterocyclic-Containing Diarylpentanoids as Bcl-2 Inhibitors Against LoVo Colorectal Cancer Cells

**DOI:** 10.3390/molecules25173877

**Published:** 2020-08-26

**Authors:** Sze Wei Leong, Suet Lin Chia, Faridah Abas, Khatijah Yusoff

**Affiliations:** 1 Department of Microbiology, Faculty of Biotechnology and Biomolecular Sciences, Universiti Putra Malaysia, UPM Serdang 43400, Selangor Darul Ehsan, Malaysia; Kyusoff@upm.edu.my; 2 UPM-MAKNA Cancer Research Laboratory, Institute of Bioscience, Universiti Putra Malaysia, UPM Serdang 43400, Selangor Darul Ehsan, Malaysia; 3 Department of Food Science, Faculty of Food Science and Technology, Universiti Putra Malaysia, UPM Serdang 43400, Selangor, Malaysia; Faridah_abas@upm.edu.my; 4 Laboratory of Natural Products, Institute of Bioscience, Universiti Putra Malaysia, UPM Serdang 43400, Selangor Darul Ehsan, Malaysia; 5 Malaysia Genome Institute (MGI), National Institute of Biotechnology Malaysia (NIBM), Jalan Bangi, Kajang 43000, Malaysia

**Keywords:** diarylpentanoids, colorectal cancer, cytotoxicity, anti-proliferative, cell cycle, apoptosis, Bcl-2 inhibitor, molecular docking

## Abstract

In the present study, we investigated the in-vitro anti-cancer potential of six diarylpentanoids against a panel of BRAF- and KRAS-mutated colorectal cancer cell lines including T84, SW620, LoVo, HT29, NCI-H508, RKO, and LS411N cells. Structure–activity relationship study suggested that the insertions of tetrahydro-4*H*-thiopyran-4-one and brominated phenyl moieties are essential for better cytotoxicity. Among the evaluated analogs, **2e** has been identified as the lead compound due to its low IC_50_ values of approximately 1 µM across all cancer cell lines and high chemotherapeutic index of 7.1. Anti-proliferative studies on LoVo cells showed that **2e** could inhibit cell proliferation and colony formations by inducing G2/M cell cycle arrest. Subsequent cell apoptosis assay confirmed that **2e** is a Bcl-2 inhibitor that could induce intrinsic cell apoptosis by creating a cellular redox imbalance through its direct inhibition on the Bcl-2 protein. Further molecular docking studies revealed that the bromophenyl moieties of **2e** could interact with the Bcl-2 surface pocket through hydrophobic interaction, while the tetrahydro-4*H*-thiopyran-4-one fragment could form additional Pi-sulfur and Pi-alkyl interactions in the same binding site. In all, the present results suggest that **2e** could be a potent lead that deserves further modification and investigation in the development of a new Bcl-2 inhibitor.

## 1. Introduction

Colorectal cancer (CRC) is the 3rd most common type of cancer and 2nd leading cause of cancer death worldwide. According to statistics by Globocan, there were approximately 1.8 million new CRC cases in 2018 and the number of CRC-related deaths has surpassed 850,000 in the same year. Worse still, these numbers are estimated to increase to 3.1 and 1.3 million cases, respectively, by 2040 [1]. To date, chemotherapy remains as the mainstream in the management of CRC. Among the currently available chemotherapy agents, oxaliplatin and 5-fluorouracil (5-FU) are the two most common drugs that have been widely used in treating CRC. Previous studies showed that the combination of these compounds could simultaneously disrupt the synthesis and the replication of DNA, thereby effectively suppressing the tumor growth [2,3]. Based on this, a mixture of oxaliplatin and 5-FU has been used as a synergistic treatment against all stages of CRC, particularly metastasis [4]. Despite such a cocktail successfully improving the CRC tumor response and prolonging the survival of CRC patients, its associated side effects including nausea, cardiotoxicity and neurotoxicity remain as issues of concern [5,6]. These undesirable side effects not only hinder the full therapeutic potential of the respective chemotherapy but also reduce the quality of life of the cancer patients. On this account, alternative effective anti-cancer agents with improved safety profile are urgently needed.

**Figure 1:**
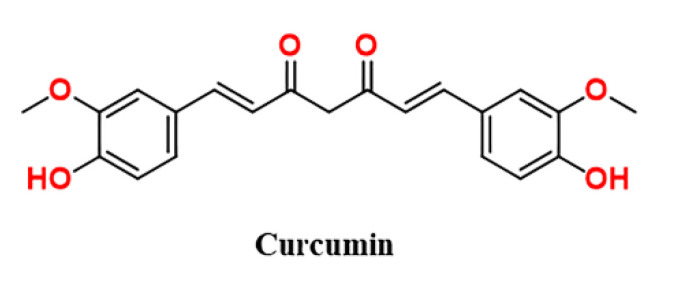
Chemical structure of curcumin.

Turmeric is a yellow powder obtained from the dried rhizome of *Curcuma longa* Linn. that has been widely used as a spice for food flavoring and coloring especially in Asian cuisines. Apart from food preparation, turmeric has also been used in traditional remedies to treat several illness and injuries including eye infections, skin rashes, indigestion, insect bites and burns [7]. Due to its medicinal values, turmeric has received a great deal of attention by researchers in the past few decades. As the major component of turmeric, curcumin (Figure 1) has been studied extensively for its biological activities and therapeutic potential. Many studies revealed that curcumin possesses strong antioxidant and inflammatory activities as it was found to scavenge several types of free radical species as well as inhibit a wide range of pro-inflammatory cytokines including tumor necrosis factor alpha (TNF-α), interleukins, cyclooxygenase-2 (COX-2) and inducible nitric oxide synthase (iNOS) [8,9,10,11]. In addition, curcumin was also found to exhibit prominent anti-cancer potential based on its growth-inhibiting and apoptosis-inducing abilities against multiple cancer cell lines [12,13,14,15]. Interestingly, this naturally occurring small molecule has displayed excellent safety profiles in both preclinical and clinical studies which prompted it to be a promising candidate in the search for a safer chemotherapeutic agent [16,17]. Unfortunately, the practical use of curcumin is limited by its low bioavailability due to the instability of the molecular structure. The β-diketone moiety of curcumin has been reported to be highly reactive and easily dissociated by several metabolic and detoxification enzymes such as aldehyde dehydrogenases and aldo-keto reductases [18,19,20]. On this account, structural optimisation to remove the unstable β-diketone moiety of curcumin has been the focus of curcuminoid-based studies.

**Figure 2:**
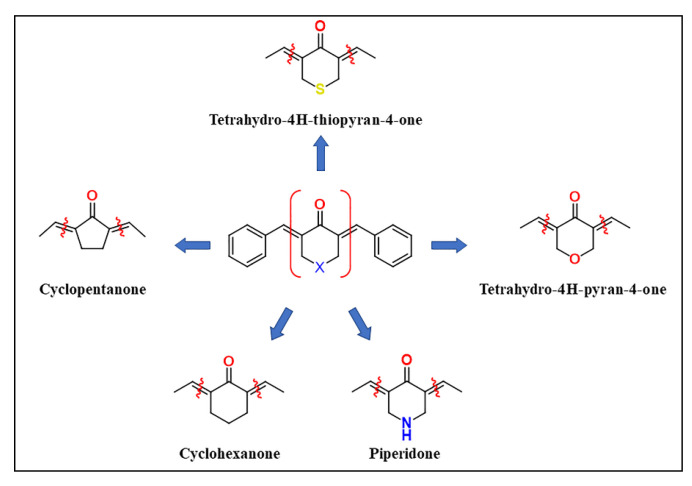
General structures of reported cyclic monocarbonyl diarylpentanoids.

Cyclic monocarbonyl diarylpentanoids are one of the bioactive families that are derived from curcumin by eliminating the reactive β-diketone moiety. Apart from showing improved chemical and metabolic stability, these diketone-free curcuminoids were also found to exhibit better medicinal potential than that of curcumin, particularly as anti-cancer agents [21,22]. Many studies reported that cyclic monocarbonyl diarylpentanoids possess potent anti-cancer properties based on their superior inhibitory activity against the growth of various cancer cell lines including breast, lung, colon and prostate cancer cells [13,23,24,25]. Several downstream mechanistic studies revealed that the remarkable anti-cancer properties of these diarylpentanoids are due to their anti-angiogenic and apoptosis-inducing activities, which resulted from VEGF inhibition and caspases activation, respectively [26,27,28]. In addition, cyclic monocarbonyl diarylpentanoids were also found to be effective in-vivo based on their positive suppression against tumor growth in several mice xenograft models [29,30]. Interestingly, recent studies demonstrated that this diarylpentanoid family possesses a curcumin-like safety profile as they were shown to be non-toxic in acute toxicity evaluations of several anti-inflammatory animal models [31,32]. Based on this, cyclic monocarbonyl diarylpentanoids are therefore of interest and worthy for further investigation in the search of safer anti-cancer drugs. Figure 2 depicts the general structures of previously reported cyclic monocarbonyl diarylpentanoids.

To the best of our knowledge, previous studies were mainly focused on the cycloalkanone, piperidone and tetrahydropyranone derivatives of diarylpentanoid as they were usually shown to possess promising anti-cancer activity. Despite the respective tetrahydrothiopyranone derivatives being rarely explored, their anti-cancer potential should not be underestimated as the cytotoxicity enhancing effects of the tetrahydropyranone moiety has recently been reported [33,34]. On the basis of this, the aim of the present study was to evaluate the anti-cancer potential of tetrahydro-4*H*-thiopyran-4-one-containing diarylpentanoids and compare to that of their respective cyclohexanone-based analogs.

## 2. Results and Discussions

### 2.1. Chemistry

**Scheme 1:**
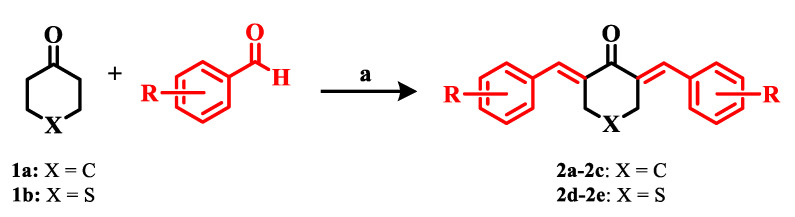
General synthetic steps for **2a**–**2e** ^x^. xReagents and conditions: (**a**) benzaldehyde, EtOH, 10 M NaOH, RT (overnight).

Scheme 1 outlines the synthetic pathway of cyclohexanone- and tetrahydro-4*H*-thiopyran-4-one-containing diarylpentanoids. As depicted, **2a**–**2e** were achieved through simple aldo-condensation by reacting cyclohexanone or tetrahydro-4*H*-thiopyran-4-one with appropriate benzaldehydes in the presence of 10 M sodium hydroxide. The formations of **2a**–**2e** were confirmed by the detection of a singlet at the chemical shift of 7.5–8.0 ppm in their respective proton NMR, where such signals represent the presence of benzylidene vinylic groups. All synthesised compounds were purified by column chromatography and characterized by ^1^H-NMR, ^13^C-NMR, and high-resolution electron impact-mass spectrometry (HRMS). The melting point of known compounds (**2a**, **2b** and **2f**) were compared to that of previously reported data [35,36,37,38]. Prior to assays, the purity of synthesized compounds was confirmed to be greater than 95% based on their respective HPLC profiles (Supplementary Materials). The synthesized compounds are listed in Table 1 and their respective chemical structures can be found in both Table 1 and Figures 9–14.

### 2.2. Biological Studies

#### 2.2.1. In-Vitro Cytotoxicity Analysis

**Figure 3:**
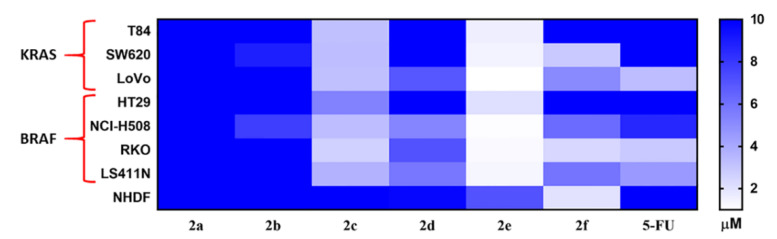
Overall IC_50_ distribution of **2a–2e** against T84, SW620, LoVo, HT29, NCI-H508, RKO, LS411N and NHDF cell lines.

The in-vitro cytotoxic activity of the synthesized compounds on seven human CRC cell lines including KRAS (T84, SW620, LoVo) and BRAF (HT29, NCI-H508, RKO and LS411N) mutated cells were determined by MTT assay as previously described [39]. The IC_50_ values obtained are summarized in Table 2 in which 5-FU and normal human dermal fibroblasts (NHDF) were used as controls. Meanwhile, a heatmap (Figure 3) was also generated using GraphPad Prism v7.0 (GraphPad Software, La Jolla, CA, USA) to visualize the cytotoxic trends of synthesized compounds graphically.

As shown in Table 2 and Figure 3, **2c** and **2e** displayed excellent cytotoxicity effects against all tested CRC cell lines (<5 µM) in which **2e** was found to be the most promising candidate based on its high chemotherapeutic index of 7.1 and low IC_50_ values ranging from 1.0 to 2.1 µM. On top of this, **2e** displayed at least 2.5-fold better cytotoxicity than that of the positive control, 5-FU, which further highlighted its potential to be developed as a chemotherapeutic agent. On the other hand, **2f** was found to be another strong cytotoxic agent as it successfully inhibited several CRC cell lines with IC_50_ values ranging from 2.4 to 6.2 µM. However, although **2f** exhibits a certain degree of anti-cancer activity, its therapeutic potential is completely offset by its undesirable cytotoxic effects on normal human cells (NHDF). Of note, all synthesized compounds displayed similar cytotoxic effects against both KRAS- and BRAF-mutated CRC cell lines, which implies that both cyclohexanone- and tetrahydro-4*H*-thiopyran-4-one-containing diarylpentanoids may not be BRAF- or KRAS-specific inhibitors. A subsequent SAR study revealed that the tetrahydro-4*H*-thiopyran-4-one fragment is crucial for improving cytotoxicity of diarylpentanoids, as both **2d** and **2e** displayed much better cytotoxicity than their respective cyclohexanone analogs (**2a** and **2b**). Interestingly, substitution of halogen at the *ortho* position of phenyl rings appeared to be essential for cytotoxicity enhancement as **2b** and **2e**, the two *ortho*-brominated diarylpentanoids, displayed stronger activity than their *ortho*-methoxylated analogs (**2a** and **2d)**. In contrast, the 3,4-difluorophenyl group was found to be undesirable as compounds with such a moiety displayed significantly higher cytotoxicity against normal human cells. These observations are consistent with our previous studies in which we reported that insertion of the *ortho*-halogenated phenyl moiety could improve the cytotoxicity of diarypentanoids while the presence of the 3,4-difluorophenyl moiety may increase compounds’ cytotoxicity against normal human cells [39,40]. Thus, in view of its potent cytotoxicity and high chemotherapeutic index, **2e** was selected for further mechanistic investigation.

#### 2.2.2. In-Vitro Anti-Proliferative Analysis

**Figure 4:**
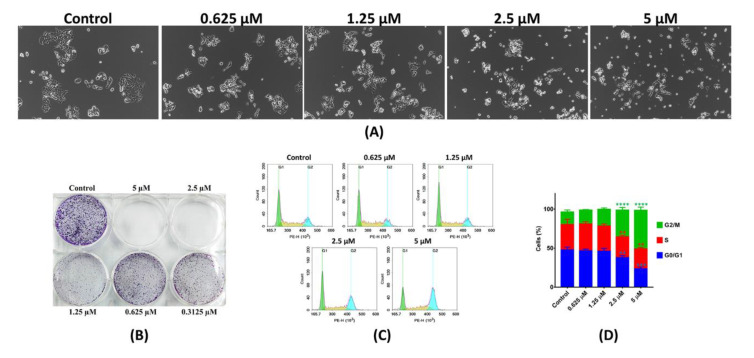
Effects of 0.625, 1.25, 2.5 and 5 µM of **2e** on cells morphology at 24 h post-treatment (**A**), colony formation at 7 days post-treatment (**B**) and cell cycle (**C** and **D**) of LoVo cells at 24 h post-treatment. * is representing the level of significant difference. The more it has, the higher the significant difference it is compared to the control.

To identify the potential cytotoxic mechanisms of **2e**, morphological inspection, colony formation and cell cycle analyses were performed. The results obtained are summarized and compiled into Figure 4.

As depicted in Figure 4A, the area of the LoVo cell islands was reduced upon the treatments with higher concentrations of **2e**, while the morphology of **2e**-treated LoVo cells was found to shrink from an epithelial-like shape to a round shape. These observations suggest that **2e** may exhibit anti-proliferative and apoptosis-inducing activities. In addition to this, **2e** was also found to inhibit the colony formation of LoVo cells in a dose-dependent manner (Figure 4B), which thereby confirmed its anti-proliferative activity. To further investigate the possible anti-proliferative mechanism, **2e** was subjected to flow cytometry-based cell cycle analysis and the results obtained are summarized in Figure 4C,D. Based on the results obtained, **2e** was found to induce G2/M-phase cell arrest in LoVo cells, as treatments with 2.5 and 5 µM of **2e** successfully increased the intensity of the G2/M peak. Thus, taking morphological inspection, colony formation and cell cycle analysis together, the present results strongly suggest that **2e** is a potent anti-proliferative agent that could induce G2/M-phase cell arrest.

#### 2.2.3. In-Vitro Cell Apoptosis Analysis

**Figure 5:**
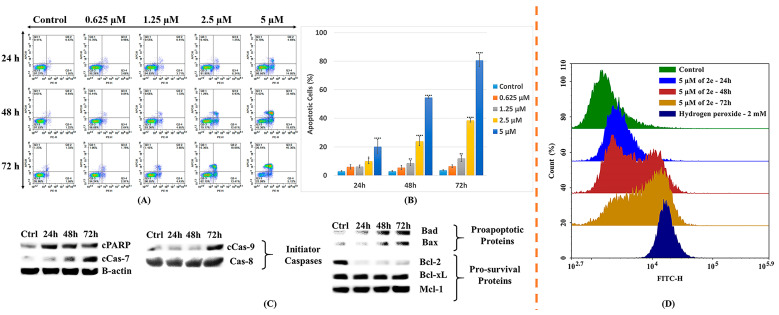
(**A**) Apoptosis profiles of LoVo cells at 24 h, 48 h and 72 h of post-treatments with 0.625, 1.25, 2.5 and 5 µM of **2e**. (**B**) Comparison of total apoptotic cells induced by **2e** at 24 h, 48 h and 72 h of post-treatments with 0.625, 1.25, 2.5 and 5 µM of **2e**. (**C**) Western blot analysis on cPARP, caspases and Bcl-2 family proteins expression in **2e**-treated (5 µM) LoVo cells at 24 h, 48 h, and 72 h of post-treatments. (**D**) Representative flow cytometry-based ROS profiles of **2e**-treated (5 µM) LoVo cells at 24 h, 48 h and 72 h of post-treatments. * is representing the level of significant difference. The more it has, the higher the significant difference it is compared to the control.

To further investigate the underlying cytotoxic mechanisms of **2e** against LoVo cells, both flow cytometry and Western blot analyses were performed. The results obtained are summarised and compiled into Figure 5.

As shown in the annexin V-based flow cytometry analysis (Figure 5A), treatment with 2.5 and 5 µM of **2e** shifted the density plots from left to right and upper right. This indicates that **2e** could induce cellular apoptosis in LoVo cells as such a shifting pattern reflects the onset and progression of cellular apoptosis. Similar to that of the cell cycle study, 0.625 and 1.25 µM of **2e** exhibited minimal insignificant effects on LoVo cells (Figure 5B), which implied that 2.5 µM could be the lowest in vitro effective dose of **2e** against LoVo cells. In order to validate the apoptosis inducing ability of **2e**, Western blot studies against cleaved PARP and cleaved caspase-7 were carried out.

As depicted in Figure 5C, both cleaved PARP and caspase-7, a hallmark of apoptotic cell death, were increased after 24 h of treatments, thus confirming the apoptotic nature of **2e** [41]. This is in consistent with that of the annexin V-based assay in which the apoptotic cells were detected as early as 24 h post-treatment. To further uncover the underlying apoptotic mechanism of **2e**, its effects on initiator caspases and proapoptotic proteins were studied. According to the results, **2e** was suggested to be an intrinsic apoptosis inducer based on its ability to activate the cleavage of caspase-9 while leaving caspase-8 unaffected (with no detection of cleaved caspase-8). This finding is further supported by the up-regulation of Bad and Bax proteins, as they are the common proteins that are actively involved in the progression of intrinsic apoptosis. However, unlike cleaved caspase-7 and cleaved PARP, the changes in Bad and Bax expression is only detectable after 48 h of post-treatment. This suggests that Bax and Bad activation might not be the primary apoptotic pathway induced by **2e**. Thus, in continuing our efforts to reveal the underlying mechanism, effects of **2e** on anti-apoptotic proteins including Bcl-2, Bcl-xL and Mcl-2 were examined. As presented in Figure 5C, Bcl-2 was significantly inhibited by **2e** as early as 24 h post-treatment, while the expressions of both Bcl-xL and Mcl-1 remained unaffected, even if the treatments were extended to 72 h. This finding suggests that Bcl-2 could be the primary target of **2e** as its down-regulation occurred earlier than the up-regulation of Bax and Bad.

In general, Bcl-2 is an anti-apoptotic protein that is well-known for its ability to prevent the permeabilisation of the mitochondrial outer membrane induced by Bak and Bax, thus protecting cells from apoptosis [42]. However, recent evidence indicates that Bcl-2 is not only an anti-apoptotic protein but also a cellular ROS regulator whereby knock-down of Bcl-2 increased subjects’ oxidative susceptibility and responses in both in-vitro and in-vivo models [43,44,45]. Based upon this, it is reasonable to suggest that Bcl-2 inhibition may lead to redox imbalance and thus increases the cellular ROS level. Therefore, with the aim to validate the role of Bcl-2 as a primary target of **2e**, we further examined the effects of **2e** on cellular ROS through DCFH-DA-based analysis.

**Figure 6:**
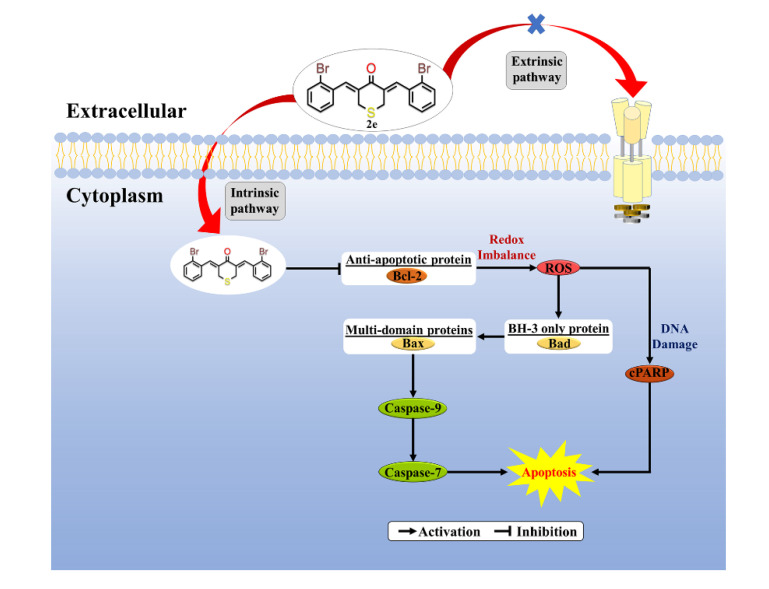
Illustration of the intrinsic apoptosis mechanism induced by **2e** in LoVo cells.

As shown in Figure 5D, 24 h of treatment with **2e** shifted the histogram to the right as a whole, implying a rapid increment in cellular ROS level. Since Bax and Bad were not activated at this time point, such an increment is therefore evidence of redox imbalance, which resulted from Bcl-2 inhibition. Based upon this, the role Bcl-2 as primary target of **2e** is thus confirmed. Of note, when the treatment was prolonged to 48 h and 72 h, an additional peak appeared at the rightmost point of the histogram, which implies a secondary increment of cellular ROS. This secondary increment could be rationalised by the stimulation of ROS, which resulted from the vicious cycle of oxidative stress induced by Bcl-2 inhibition. Taken together, the present results confirmed that **2e** is a Bcl-2 inhibitor that can induce intrinsic apoptosis by disrupting the cellular redox state. The overall apoptosis mechanism induced by **2e** is summarised in Figure 6.

#### 2.2.4. In-Silico Molecular Modelling Analysis

In order to gain further insights into the binding mode of **2e** in Bcl-2, flexible docking was performed using Discovery Studio 3.1. Prior to docking **2e** into Bcl-2, the docking parameters were first validated by the re-docking of the co-crystallised ligand into the active site of the protein. The parameters are considered acceptable if the root mean square deviation (RMSD) value obtained from the re-docked and native ligands does not exceed 2.0 Å [46].

**Figure 7:**
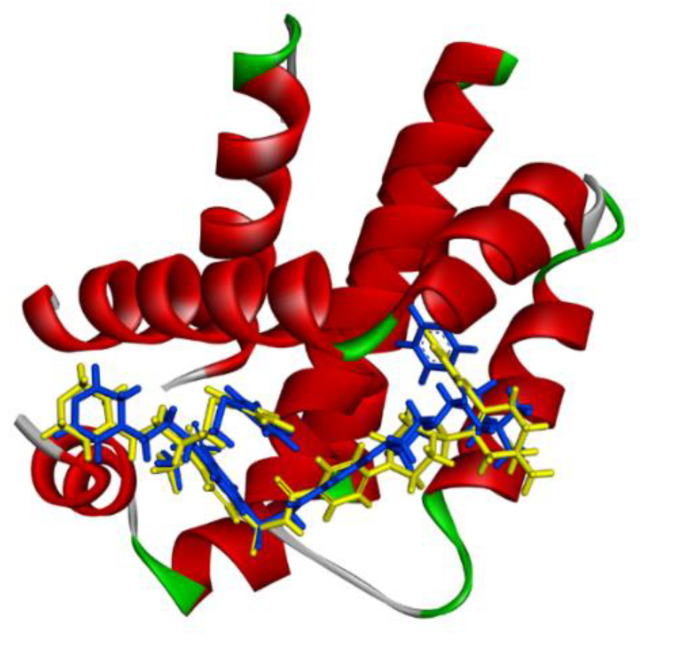
Overlay of re-docked (yellow) and native (blue) conformations of ABT-263 in Bcl-2 (PDB ID: 4LVT).

**Figure 8:**
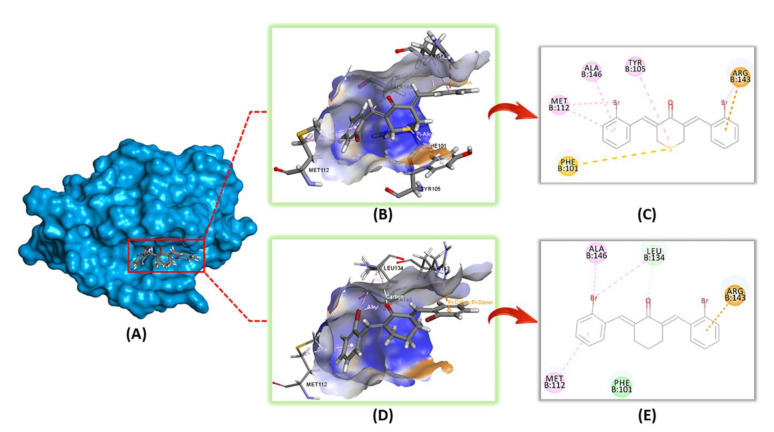
Molecular docking model of **2e** with the Bcl-2 protein (PDB: 4LVT). (**A**) 3D conformation positions of **2b** and **2e** in the surface pocket of Bcl-2. (**B**) Key interactions of **2e** in the surface pocket of Bcl-2. (**C**) 2D graph of **2e** in the surface pocket of Bcl-2. (**D**) Key interactions of **2b** at the surface pocket of Bcl-2. (**E**) 2D graph of **2b** in the surface pocket of Bcl-2.

As depicted in Figure 7, both redocked (yellow) and native (blue) ABT-263 were found to interact with Bcl-2 in very similar conformations, of which the respective RMSD value obtained was 1.8 Å. This implies that the selected parameters are acceptable and could be used further for the docking studies of **2e**. In order to compare and contrast the binding interactions contributed by tetrahydro-4*H*-thiopyran-4-one and cyclohexanone fragments, **2b** was also included in the present docking analysis. Figure 8 illustrates the binding interactions of **2b** and **2e** in Bcl-2.

As shown in Figure 8A, both **2b** and **2e** bound to the surface pocket of bcl-2 protein in very similar conformations. This implies that **2b** and **2e** might share some similarity in interacting with the Bcl-2 protein. A closer look at their respective binding interactions (Figure 8B–D) shows that the bromophenyl fragments of both compounds could interact with the Bcl-2 surface pocket by forming hydrophobic interactions with several amino acid residues including ALA-146, MET-112 and ARG-143. This rationalises the increased cytotoxicity seen by brominated analogs, as the bromo group is more hydrophobic than methoxyl moiety. More importantly, the present docking analysis revealed that the thio-moiety of **2e** could form additional Pi-sulfur and Pi-alkyl interactions with PHE-101 and TYR-105 residues, respectively, thus explaining the improved cytotoxicity of tetetrahydro-4*H*-thiopyran-4-one-containing analogs. The in silico molecular docking results are summarized in Table 3.

## 3. Materials and Methods

### 3.1. Chemistry

Chemicals, reagents and solvents were purchased from Sigma-Aldrich and Merck, and were used without purification. The chemical reactions were routinely checked on 0.20 mm Merck TLC plate silica gel 60 F254 using a solvent system of ethyl acetate/hexane (15:85). Compound purifications were performed using column chromatography on Merck silica gel 60 (mesh 70–230). Melting points were determined using the Fisher–Johns melting point apparatus and were uncorrected. High-resolution electron ionisation-mass spectrometry (HREI-MS) was determined using a DFS high resolution GC/MS (Thermo Scientific, San Jose, CA, USA). Nuclear magnetic resonance spectra were recorded on a Varian 500 MHz NMR Spectrometer.

#### General Procedure for the Synthesis of 2a–2e

**Figure 9:**
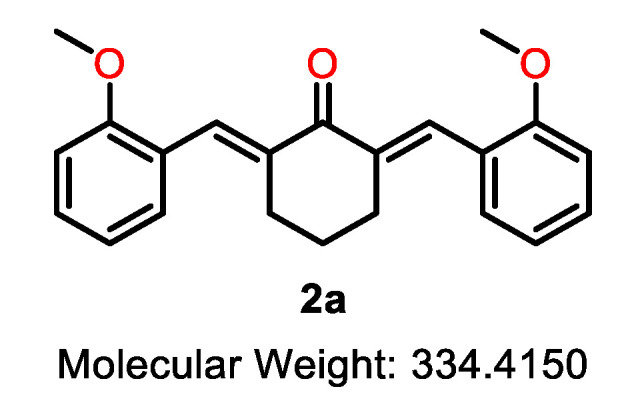
Chemical structure of **2a**.

**Figure 10:**
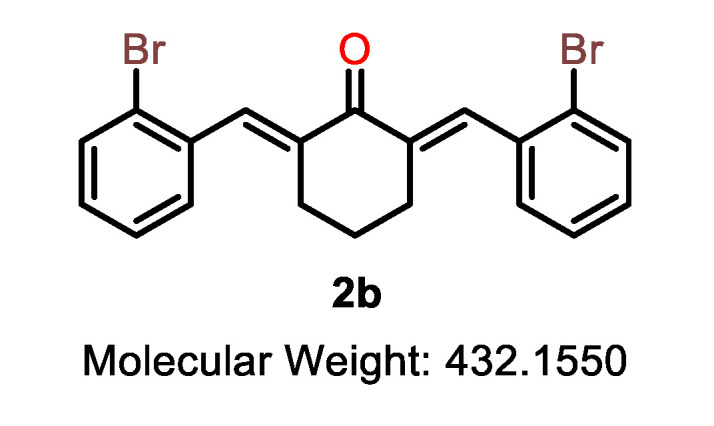
Chemical structure of **2b**.

**Figure 11:**
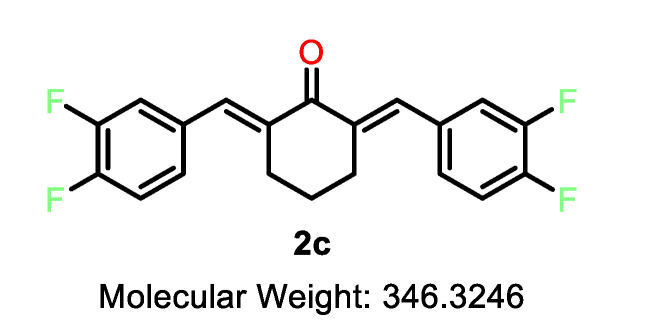
Chemical structure of **2c**.

**Figure 12:**
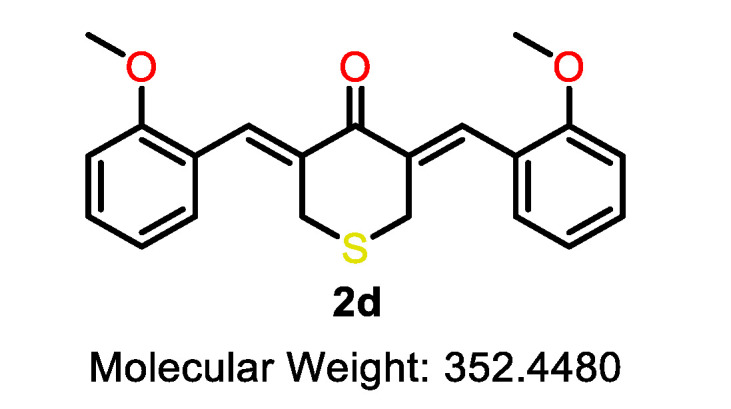
Chemical structure of **2d**.

**Figure 13:**
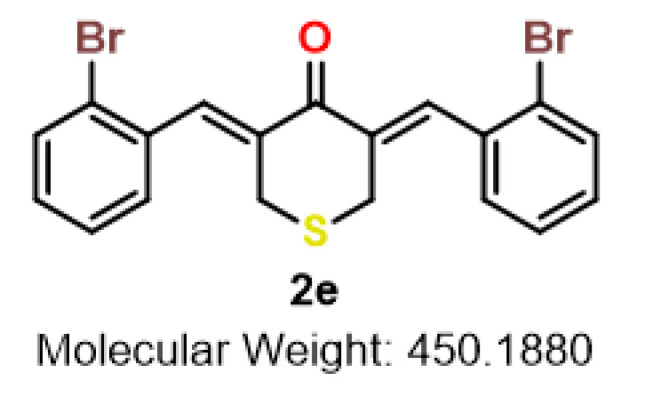
Chemical structure of **2e**.

**Figure 14:**
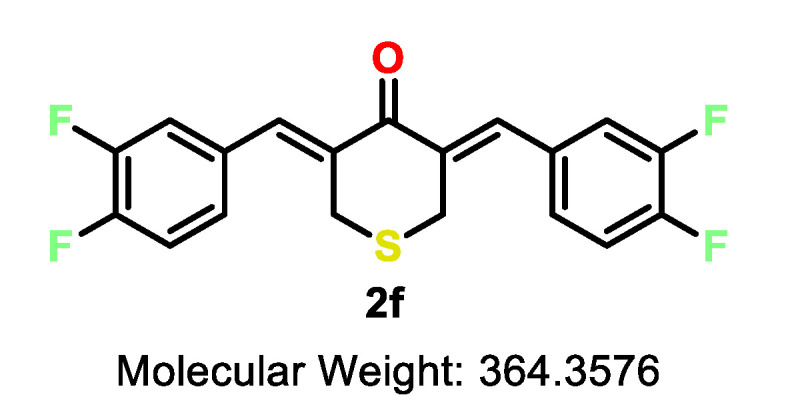
Chemical structure of **2f**.

Cyclohexanone or tetrahydro-4*H*-thiopyran-4-one (1 mmol) and appropriate aromatic aldehydes (2 mmol) were reacted overnight in 20 mL of absolute ethanol in the presence of a catalytic amount of 10 M NaOH aqueous solution. Then, the reaction mixture was poured into a 250 mL beaker containing 100 mL of 3 M HCl followed by extraction with ethyl acetate. The ethyl acetate layer was collected and dried over anhydrous magnesium sulfate followed by evaporation with a rotatory evaporator to give the crude of the desired compounds. Purifications with chromatography using an ethyl acetate/hexane (10:90) solvent system were then performed to obtain pure diarylpentanoids (Figure 9, Figure 10, Figure 11, Figure 12, Figure 13 and Figure 14).

*2,6-Bis(2-methoxybenzylidene)cyclohexanone* (**2a**). (Figure 9). Yellow gummy solid; yield: 87%; m.p. found: m.p. reported: Mass calculated: 334.1569; Mass found: 334.1588. ^1^H NMR (500 MHz, chloroform-*d*) δ ppm 1.74 (*d*_t_, *J* = 12.21, 6.24 Hz, 2 H) 2.84 (t, *J* = 5.49 Hz, 4 H) 3.85 (s, 6 H) 6.91 (d, *J* = 8.23 Hz, 2 H) 6.96 (t, *J* = 7.41 Hz, 2 H) 7.28–7.35 (m, 4 H) 7.98 (s, 2 H). ^13^C NMR (126 MHz, chloroform-*d*) δ ppm 23.5, 28.8, 55.5, 110.6, 119.9, 125.1, 123.0, 130.3, 132.5, 136.5, 158.3, 190.6.

*2,6-Bis(2-bromobenzylidene)cyclohexanone* (**2b**) (Figure 10). Yellow solid; yield: 97 %; m.p. found: 106–107 °C; m.p. reported: 120 °C; Mass calculated: 429.9568; Mass found: 429.9585. ^1^H NMR (500 MHz, chloroform-*d*) δ ppm 1.76 (quin, *J* = 6.04 Hz, 2 H) 2.75 (t, *J* = 5.21 Hz, 4 H) 7.17–7.22 (m, 2 H) 7.29–7.35 (m, 4 H) 7.64 (d, *J* = 8.23 Hz, 2 H) 7.85 (s, 2 H). ^13^C NMR (126 MHz, chloroform-*d*) δ ppm 23.1, 28.3, 110.0, 125.1, 126.9, 129.7, 130.5, 132.9, 136.2, 136.4, 137.4, 189.6

*2,6-Bis(3,4-difluorobenzylidene)cyclohexanone* (**2c**) (Figure 11). Brown solid; yield: 43%; m.p.: 90–91 °C; Mass calculated: 346.0981; Mass found: 346.1094. ^1^H NMR (500 MHz, chloroform-*d*) δ ppm 1.82 (quin, *J* = 6.18 Hz, 2 H) 2.89 (t, *J* = 5.21 Hz, 4 H) 7.15–7.23 (m, 4 H) 7.25–7.32 (m, 2 H) 7.67 (s, 2 H). ^13^C NMR (126 MHz, chloroform-*d*) d ppm 22.62, 28.19, 117.32, 117.46, 118.75, 118.88, 126.98, 127.00, 127.02, 127.05, 132.76, 132.79, 132.80, 132.83, 134.97, 136.45, 136.47, 149.04, 149.15, 149.24, 149.34, 151.02, 151.12, 151.25, 151.35, 189.6

*3,5-Bis(2-methoxybenzylidene)tetrahydro-4H-thiopyran-4-one* (**2d**) (Figure 12). Pale yellow solid; yield: 83 %; m.p.: 120–121 °C; Mass calculated: 352.1133; Mass found: 352.1145. ^1^H NMR (500 MHz, chloroform-*d*) δ ppm 3.81 (s, 4 H) 3.86 (s, 6 H) 6.93 (d, *J*=8.23 Hz, 2 H) 6.97 (t, *J* = 7.41 Hz, 2 H) 7.20–7.28 (m, 2 H) 7.34 (t, *J* = 7.96 Hz, 2 H) 7.91 (s, 2 H). ^13^C NMR (126 MHz, chloroform-*d*) d ppm 30.3, 55.5, 110.8, 120.1, 124.3, 130.4, 130.4, 132.9, 134.3, 158.2, 189.3

*3,5-Bis(2-bromobenzylidene)tetrahydro-4H-thiopyran-4-one* (**2e**) (Figure 13). Pale yellow solid; yield: 78 %; m.p.: 158–160 °C; Mass calculated: 447.9132; Mass found: 447.9140. ^1^H NMR (500 MHz, chloroform-*d*) δ ppm 3.73 (s, 4 H) 7.20–7.25 (m, 2 H) 7.27 (d, *J* = 5.49 Hz, 2 H) 7.32–7.37 (m, 2 H) 7.65 (d, *J* = 8.23 Hz, 2 H) 7.80 (s, 2 H). ^13^C NMR (126 MHz, chloroform-*d*) δ ppm 29.7, 124.8, 127.2, 130.1, 130.4, 133.2, 135.1, 135.5, 136.3, 188.5

*3,5-Bis(3,4-difluorobenzylidene)tetrahydro-4H-thiopyran-4-one* (**2f**) (Figure 14). Pale yellow solid; yield: 55%; m.p. found: 124–125 °C; m.p. reported: 135–137 °C; Mass calculated: 364.0545; Mass found: 364.0558. ^1^ H NMR (500 MHz, chloroform-*d*) δ ppm 3.87 (s, 4 H) 7.14 (dd, *J* = 8.23, 2.20 Hz, 2 H) 7.19–7.28 (m, 4 H) 7.66 (s, 2 H). ^13^C NMR (126 MHz, chloroform-*d*) δ ppm 29.88, 76.76, 77.01, 77.27, 117.65, 117.78, 118.67, 118.81, 126.57, 126.60, 126.62, 126.65, 131.88, 131.91, 131.92, 134.25, 134.26, 134.82, 149.18, 149.27, 149.49, 149.58, 151.16, 151.26, 151.50, 151.60, 188.21.

### 3.2. Biological Evaluations

#### 3.2.1. Cell Culture

A panel of KRAS (T84, SW620 and LoVo) and BRAF (HT29, NCI-H508, RKO and LS411N) mutated human CRC cell lines were grown in Dulbecco’s modified Eagle’s medium (DMEM) containing 10% fetal bovine serum (FBS) and 1% penicillin/streptomycin at 37 °C under 5% CO_2_ while NHDF cells were cultured in DMEM with 5% FBS under the same condition. The culture medium was replenished every two days until the cells reached 80–90% confluency for further usage. All cell lines (T84, ATCC^®^CCL-248™; SW620, ATCC^®^CCL-227™; LoVo, ATCC^®^CCL-229™; HT-29, ATCC^®^HTB-38; NCI-H508, ATCC^®^CCL-253™; RKO, ATCC^®^CRL-2577; LS411N, ATCC^®^CRL-2159; NHDF, ATCC^®^ PCS-201-010™) were purchased from American Type culture Collections (ATCC, Manassas, VA, USA).

#### 3.2.2. MTT Assay

LoVo cells (1.5–5 × 10^3^ cells/well) were seeded into a 96 well plate and incubated at 37 °C and 5% CO_2_ atmosphere for 24 h. On the following day, the cell media were replaced with 100 µL of test compounds at various concentration. The final concentration of DMSO in the well is 0.1%. After 72 h of incubation, culture medium was removed and 100 µL of MTT (thiazoyl blue tertrazolium bromide) solution (0.5 mg/mL in culture media) was added. The resultant plates were then further incubated at 37 °C for 4 h. Upon completion, the MTT solution was removed and the formazan salts formed were dissolved in 100 µL of DMSO. The absorbance was recorded with a microplate reader at 570 nm. The IC_50_ values of tested compounds were calculated from three independent experiments.

#### 3.2.3. Morphological Inspection

LoVo cells (5 × 10^4^ cells/well) were seeded into a 12 well plate and incubated at 37 °C and 5% CO_2_ atmosphere for 24 h. Next, the culture media were replaced with 1 mL of test compounds at various concentration and further incubated for 24 h. The final concentration of DMSO in the well is 0.1%. The morphological changes of compound-treated cells were photographed using a fluorescent microscope.

#### 3.2.4. Colony Formation Assay

LoVo cells (1 × 10^3^ cells/well) were seeded into a 6-well plate and incubated at 37 °C and 5% CO_2_ atmosphere overnight. After incubation, the culture media were replaced with 3 mL of test compounds at various concentration and further incubated for seven days. Upon completion, cells were washed with PBS and fixed with 4% paraformaldehyde for 15 min followed by cell staining with 0.5% (*w*/*v*) crystal violet for 20 min. The resultant plates were then washed with tap water and dried at room temperature. Upon drying, the plate image was photographed.

#### 3.2.5. Cell Cycle Analysis

LoVo cells (1 × 10^5^ cells/well) were seeded into a 6-well plate and incubated at 37 °C and 5% CO_2_ atmosphere overnight. The seeded cells were then treated with different concentrations of tested compounds for 24 h. Upon completion, cells were trypsinized and washed with PBS followed by fixation with 70% ethanol overnight at 4 °C. Then, the fixed cells were centrifuged at 160× *g* for 5 min and the resultant cell pellet was washed twice with PBS. Subsequently, 20 μL of DNAse-free RNAse A solution (1 mg/mL) was added to the cell-containing PBS and further incubated for 15 min. Upon completion, 10 μL of propidium iodide staining buffer (10 mg/mL) was added and the resulting mixture was further incubated for 20 min at 4 °C in the dark. Lastly, samples were analyzed with a flow-cytometer (ACEA Biosciences, Inc., San Diego, CA, USA) at the PE channel. The values presented are the means ± SD of three independent experiments.

#### 3.2.6. Cell Apoptosis Analysis

LoVo cells (1 × 10^5^ cells/well) were seeded into a 6-well plate and incubated at 37 °C and 5% CO_2_ atmosphere overnight. The seeded cells were then treated with different concentrations of tested compounds for 24, 48 and 72 h. After treatments, cells were trypsinized and washed with PBS followed by the addition of 1× binding buffer (500 μL), Annexin V-PE (5 μL) and 7-AAD (5 μL) solutions. The resulting mixture was then incubated in the dark at 4 °C for 20 min. Upon completion, the mixture was analysed with a flow-cytometer at the PE and APC channel. The values presented are the means ± SD of three independent experiments.

#### 3.2.7. Measurement of Intracellular Reactive Oxygen Species (ROS) Levels

LoVo cells (1 × 10^5^ cells/well) were seeded into a 6-well plate and incubated at 37 °C and 5% CO_2_ atmosphere overnight. After incubation, the seeded cells were treated with 1 mL of 10 µM DCFDA in culturing media for 1 h. Upon completion, the cells were washed twice with PBS followed by treatments with 5 µM of selected compounds for 24, 48 and 72 h. Then, the treated cells were harvested, washed and re-suspended in PBS followed by flow-cytometric analysis at the FITC channel.

#### 3.2.8. Western Blot Analyses

Lovo cells (1.5 × 10^4^ cells/cm^2^) were seeded in a T75 flask and incubated at 37 °C and 5% CO_2_ atmosphere overnight. The seeded cells were then treated with selected compounds at 5 µM of **2e** for 24 h, 48 h, and 72 h. After treatment, the cells were harvested and lysed with phenylmethanesulfonyl fluoride (PMSF)-containing cell lysis buffer. The lysates were then centrifuged at 13,300× *g* for 15 min and the supernatant was collected. Protein concentrations of all samples were determined using a BCA assay kit (Thermo Scientific Inc., Waltham, MA, USA). Equal amounts of protein were electrophoresed in 12% and 20% SDS-polyacrylamide gel (SDS-PAGE), and electro-transferred onto a 0.22-μm polyvinyldene difluoride membrane. Each membrane was blocked with 5% (*w*/*v*) skim milk at room temperature for 1 h followed by overnight incubation with specific primary antibodies (1:1000) at 4 °C. Subsequently, the respective membrane was washed thrice with TBST (0.05% (*v*/*v*) of tween-20) followed by 1 h incubation with secondary antibody (1:10,000). Upon completion, the membrane was washed thrice with 1X Tris-Buffered Saline, 0.1% Tween^®^ 20 Detergent (TBST) and the immune reactive bands were developed using SuperSignal West Dura Extended Duration Substrate (Thermo Scientific Inc., Waltham, MA, USA). Subsequently the blots were visualized with an Amersham Imager 600 system (GE Healthcare Bio-Sciences AB, Uppsala, Sweden). β-actin served as an internal control [47]. All primary antibodies (cPARP, #5625; cCas-7, #12827; B-actin, #4967; cCas-9, #9508; Cas-8, #9746; Bad, #9239; Bax, #5023; Mcl-1, #94296) except anti-Bcl-2 and anti-Bcl-xL were purchased from Cell signaling Technology (Beverly, MA, USA). Both anti-Bcl-2 (K200018M) and anti-Bcl-xL (K001595P) primary antibodies were purchased from Solarbio Life Sciences (Beijing, China).

#### 3.2.9. Molecular Docking

Molecular docking studies were carried out using Discovery Studio 3.1 (Accelrys, San Diego, USA) on an Intel^®^ (TM)2 Quad CPU Q8200 @2.33 GHz running under a Windows XP Professional environment.

(1) Receptor preparation

The crystal structure of Bcl-2 (PDB ID: 4LVT) was first obtained from the Protein Data Bank. Then, all water molecules and co-crystallized ligands were removed from the crystal structure to obtain the Bcl-2 receptor. Prior to docking the selected compounds into Bcl-2, the Bcl-2 receptor underwent a protein preparation protocol with CHARMm force field.

(2) Ligand preparation

Co-crystallized ligand (ABT-263) and **2e** were drawn with ChemDraw Ultra 12.0. Then, the structures were imported to the Discovery Studio 3.1 followed by the ligand preparation protocol with the default setting recommended by Accelrys. The prepared ligands were then subjected to ligand minimization with CHARMm force field before being used for docking analyses.

(3) Flexible docking

Minimized co-crystallized ligands were re-docked into their respective enzymes with several sets of amino acids as flexible residues. The top ranked conformations resulted from the docking experiment were compared to their original crystallographic confirmation in terms of RMSD. The parameters with lowest RMSD values were selected for the flexible docking of **2e**. The flexible docking results were analyzed using Discovery Studio Visualizer v4.1.0.14169 (Accelrys, San Diego, CA, USA).

## 4. Conclusions

In summary, six diarylpentanoids were synthesized and investigated for their anti-cancer potential against a panel of KRAS- and BRAF-mutated CRC cell lines. Compound **2e** was found to be the most potent candidate based on its low IC_50_ values and high chemotherapeutic index. The anti-proliferative study on LoVo cells shows that **2e** could inhibit cell proliferation by triggering cell cycle arrest at the G2/M phase. Further cell apoptosis analysis revealed that **2e** could induce intrinsic apoptosis by disrupting the cellular redox balance through its direct inhibitory effects on Bcl-2 protein. A subsequent molecular docking study suggested that the insertion of the thio group into the cycloalkane ring and the substitution of a hydrophobic moiety on the phenyl ring could improve the binding interactions between Bcl-2 and diarylpentanoids, thus leading to better anti-cancer activity. In conclusion, we believe that tetrahydro-4H-thiopyran-4-one-containing diarylpentanoids could be a new class of Bcl-2 inhibitor that deserves further investigation in the search for new anti-cancer agents.

## Figures and Tables

**Table 1:** Chemical Structures of **2a**–**2e.**

Cmpd	Structure	Cmpd	Structure	Cmpd	Structure
**2a**	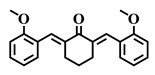	**2b**	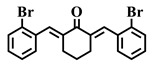	**2c**	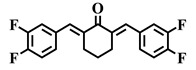
**2d**	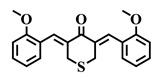	**2e**	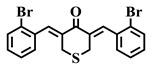	**2f**	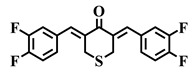

Cmpd = Compound.

**Table 2:**
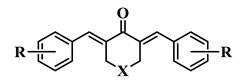
Cytotoxicity of **2a**–**2e** on T84, SW620, LoVo, HT29, NCI-H508, RKO, LS411N and normal human dermal fibroblast (NHDF) cell lines at 72 h post-treatment.

Compound	X	R	IC_50_ (µM)							NHDF	Chemo- Therapeutic Index
			KRAS			BRAF					
			T84	SW620	LoVo	HT29	NCI-H508	RKO	LS411N		
5-FU	-	-	>10	>10	3.3	>10	8.6	2.9	4.6	>20	-
2a	C	2-OMe	>10	>10	>10	>10	>10	>10	>10	>20	-
2b	C	2-Br	>10	8.9	>10	>10	7.8	>10	>10	>20	-
2c	C	3,4-F	3.3	3.2	3.3	5.4	3.3	2.6	3.7	12.9	2.3–5.0
2d	S	2-OMe	>10	>10	6.9	>10	5.3	7.1	5.8	9.7	1.4–1.8
2e	S	2-Br	1.6	1.4	1.0	2.1	1.1	1.2	1.3	7.1	3.4–7.1
2f	S	3,4-F	>10	2.9	5.1	>10	6.2	2.4	5.9	2.0	0.3–0.8

**Table 3:** Data resulted from the molecular docking of **2b** and **2e** in Bcl-2.

Compound	Structure	Interacting Amino Acid Residue	Type of Interaction	Bonding Distance (Å)
2b	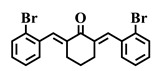	ALA-146	Alkyl	3.84
		MET-112	Pi-Alkyl	4.12
		ARG-143	Pi-Cation	2.39
		LEU-134	Alkyl	4.8
			Carbon	2.49
2e	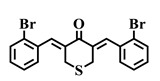	ALA-146	Pi-Alkyl	5.20
		MET-112	Pi-Alkyl	4.76
			Alkyl	4.75
		ARG-143	Alkyl	3.93
			Pi-Cation	3.67
			Pi-Alkyl	5.14
		TYR-105	Pi-Alkyl	5.24
		PHE-101	Pi-Sulfur	4.78

## Supplementary Materials

The following are available online. Figure S1: NMR Spectral and HPLC Profile of Synthesized Compound 2a; Figure S2: NMR Spectral and HPLC Profile of Synthesized Compound 2b; Figure S3: NMR Spectral and HPLC Profile of Synthesized Compound 2c; Figure S4: NMR Spectral and HPLC Profile of Synthesized Compound 2e; FigureS5: NMR Spectral and HPLC Profile of Synthesized Compound 2f.
