# Supplementary material for: In-Vitro and In-Silico Evaluations of Heterocyclic-Containing Diarylpentanoids as Bcl-2 Inhibitors Against LoVo Colorectal Cancer Cells
Source: Molecules. 2020 Aug 26;25(17):3877. doi: 10.3390/molecules25173877 (PMC7504466; doi:10.3390/molecules25173877)
Supplement: Supplementary file 1 [file molecules-25-03877-s001.pdf]

Supplementary Material

In-vitro and *in-silico* evaluations of heterocyclic-containing diarylpentanoids as Bcl-2 inhibitor against LoVo colorectal cancer cells

Sze Wei Leong [1]*, Suet Lin Chia [1,2]*, Faridah Abas [2,3], Khatijah Yusoff [1,2,4]

[1] Department of Microbiology, Faculty of Biotechnology and Biomolecular Sciences, Universiti Putra Malaysia, 43400 UPM Serdang, Selangor Darul Ehsan, Malaysia; Frederick_leong@hotmail.com (SW.L); suetlin@upm.edu.my (SL.C.); Kyusoff@upm.edu.my (K.Y)
[2] Institute of Bioscience, Universiti Putra Malaysia, 43400 UPM Serdang, Selangor Darul Ehsan, Malaysia; Faridah_abas@upm.edu.my (F.A)
[3] Department of Food Science, Faculty of Food Science and Technology, Universiti Putra Malaysia, 43400 UPM Serdang, Selangor, Malaysia
[4] Malaysia Genome Institute (MGI), National Institute of Biotechnology Malaysia (NIBM), Jalan Bangi, 43000 Kajang, Selangor Darul Ehsan, Malaysia
* Correspondence: Frederick_leong@hotmail.com; Tel.: +603- 9769 7765 (SW.L); suetlin@upm.edu.my; Tel.: +603- 9769 8295 (SL.C)

*Corresponding authors

Tel +603-89467765; Fax: +603-89467590

E-mail address: frederick_leong@hotmail.com (SW. L), suetlin@upm.edu.my (SL.C)

NMR Spectral and HPLC Profile of Synthesized Compounds

Compound **2a**

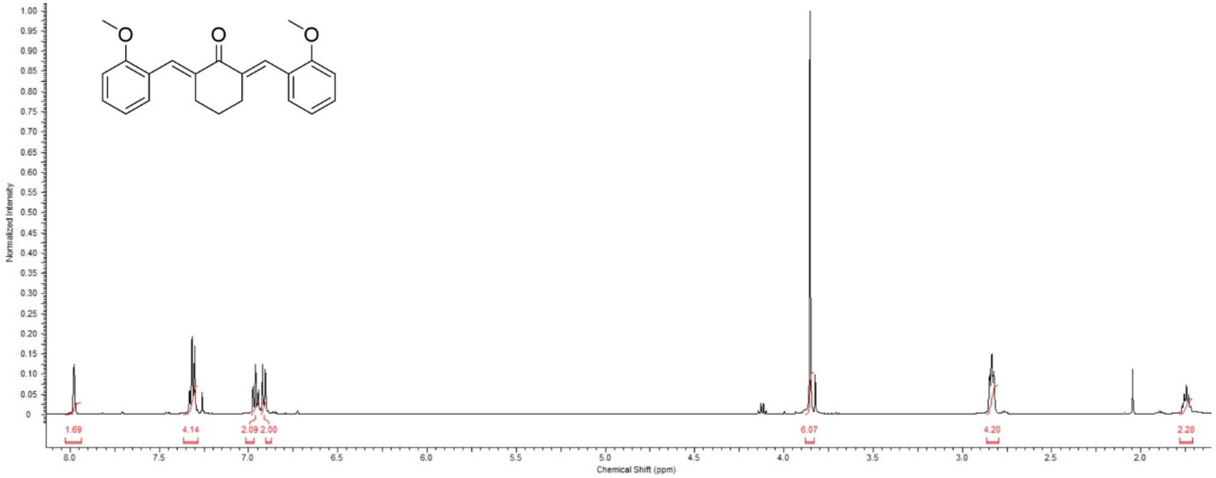

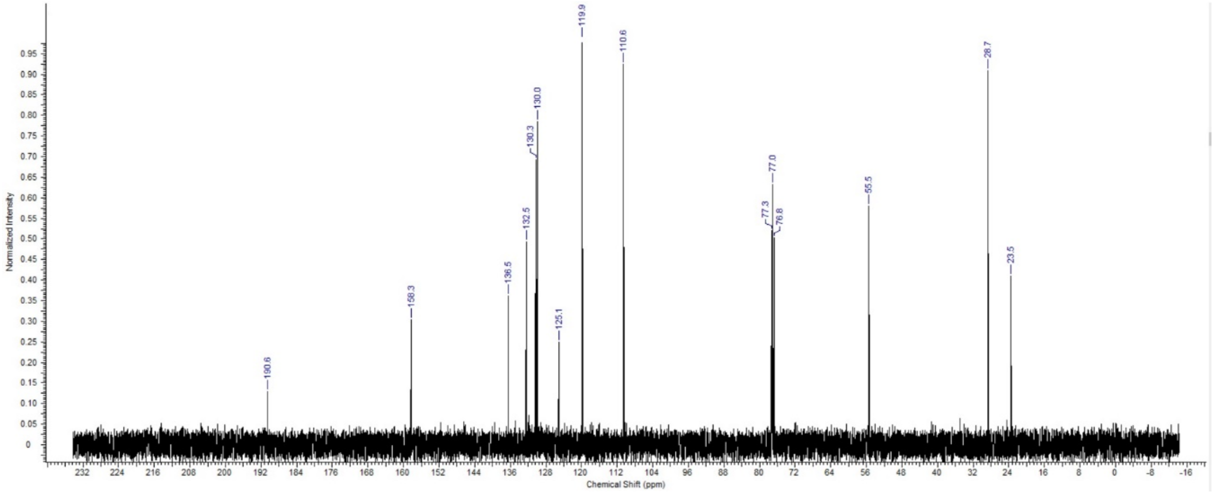

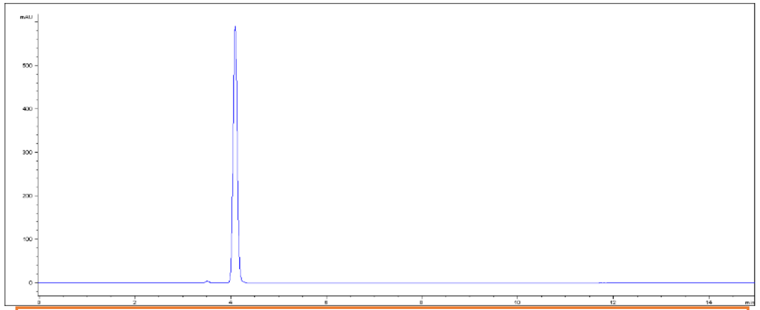

HPLC Conditions:
Column: Thermohypersil C18 Gold (4.6mm ID X 150mm)
Injection volume: 5 µL
flowrate: 1mL/min
Isocratic with 30% water: 70% ACN
Wavelength: 360nm
Retention Time: 4 minutes

Compound 2b

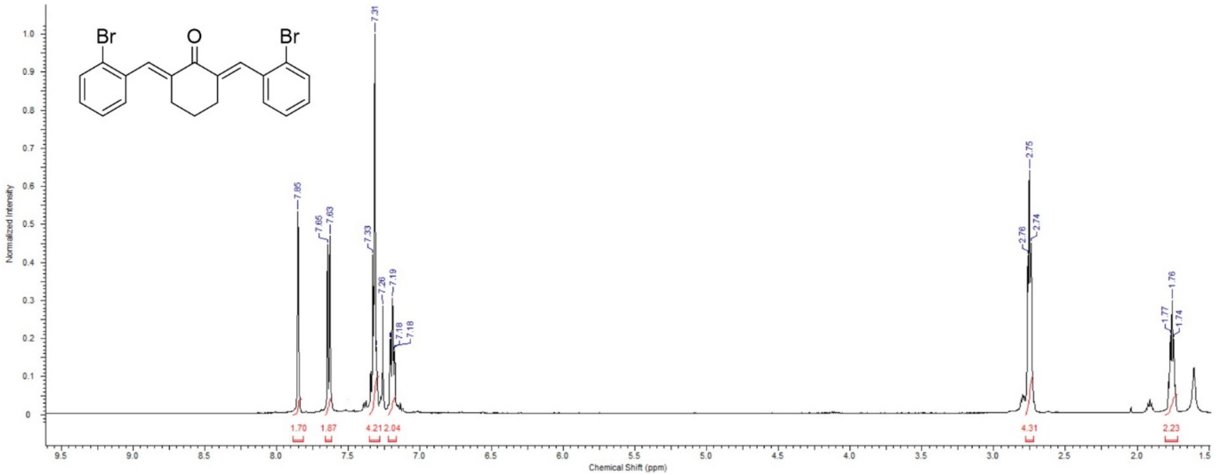

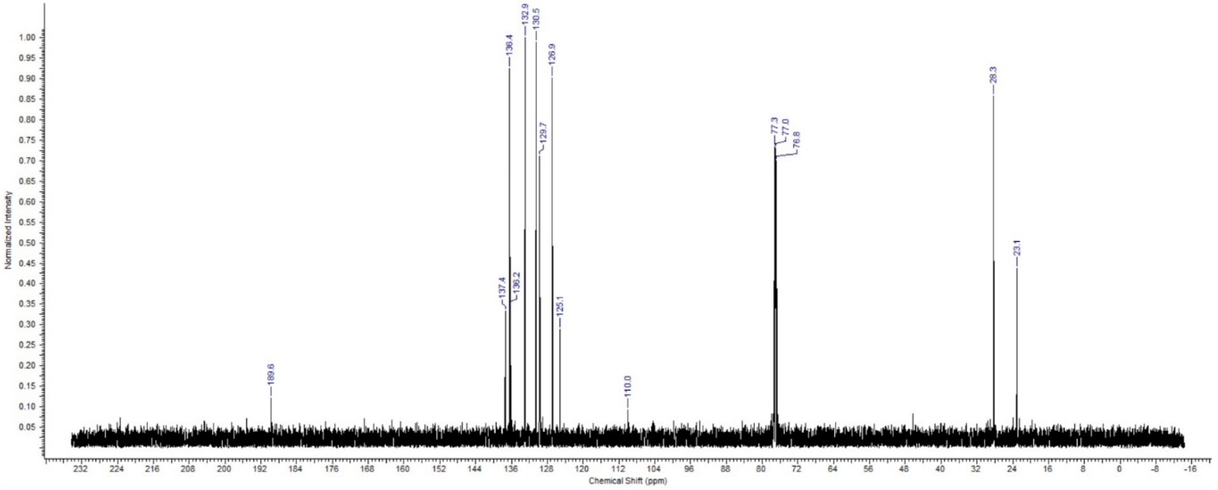

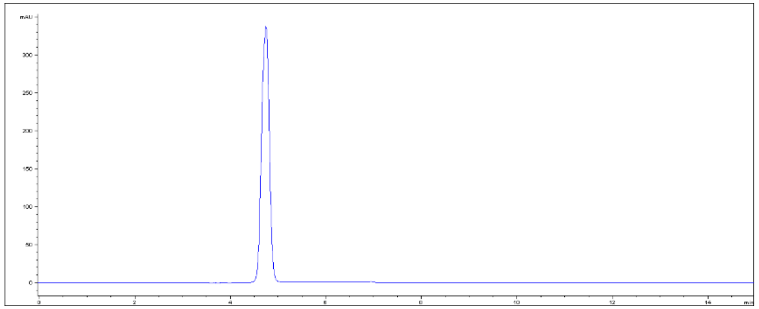

HPLC Conditions:
Column: Thermohypersil C18 Gold (4.6mm ID X 150mm)
Injection volume: 5 µL
flowrate: 1mL/min
Isocratic with 30% water: 70% ACN
Wavelength: 360nm
Retention time: 4.75 minutes

Compound 2c

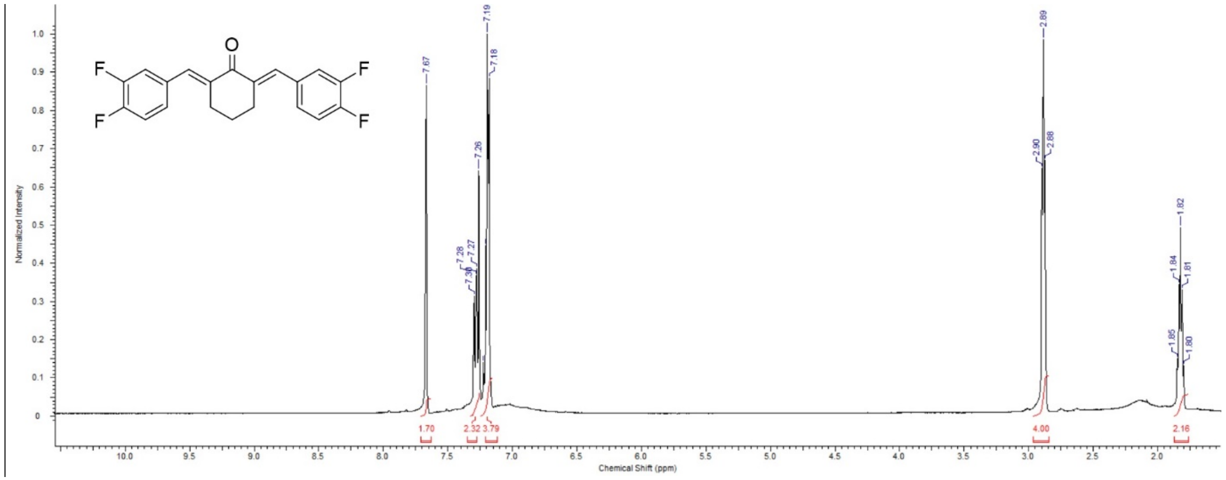

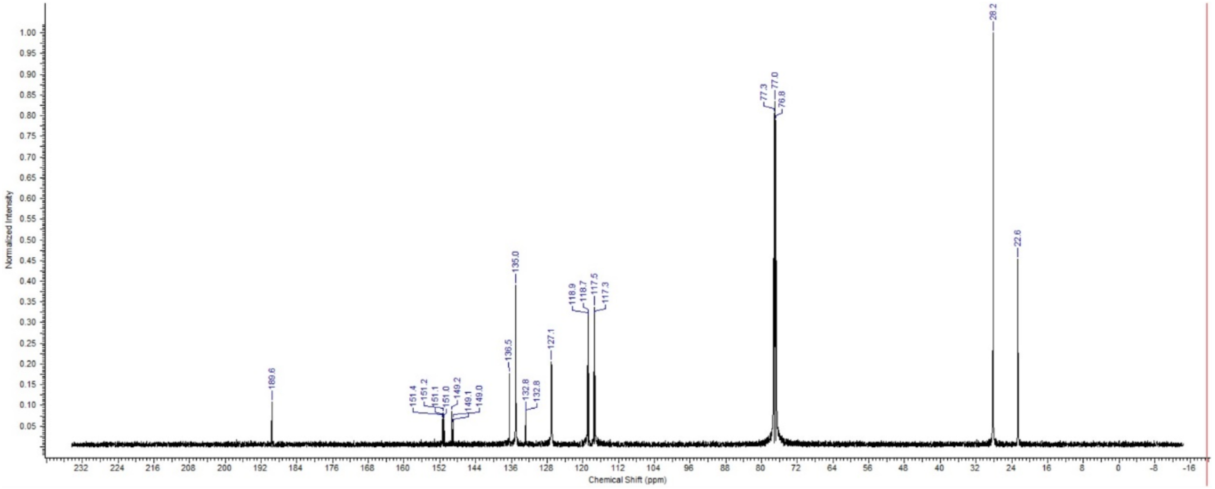

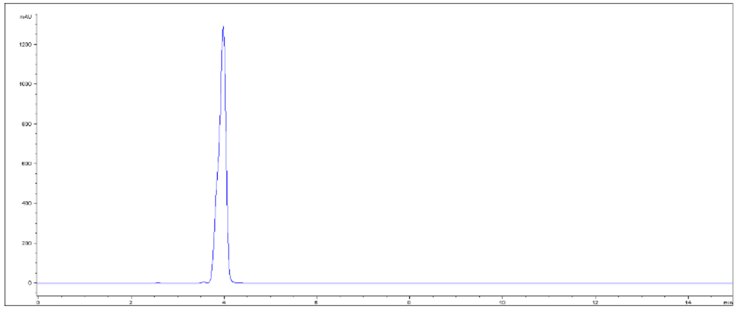

HPLC Conditions:
Column: Thermohypersil C18 Gold (4.6mm ID X 150mm)
Injection volume: 5 µL
flowrate: 1mL/min
Isocratic with 30% water: 70% ACN
Wavelength: 360nm
Retention Time: 4 minutes

Compound 2d

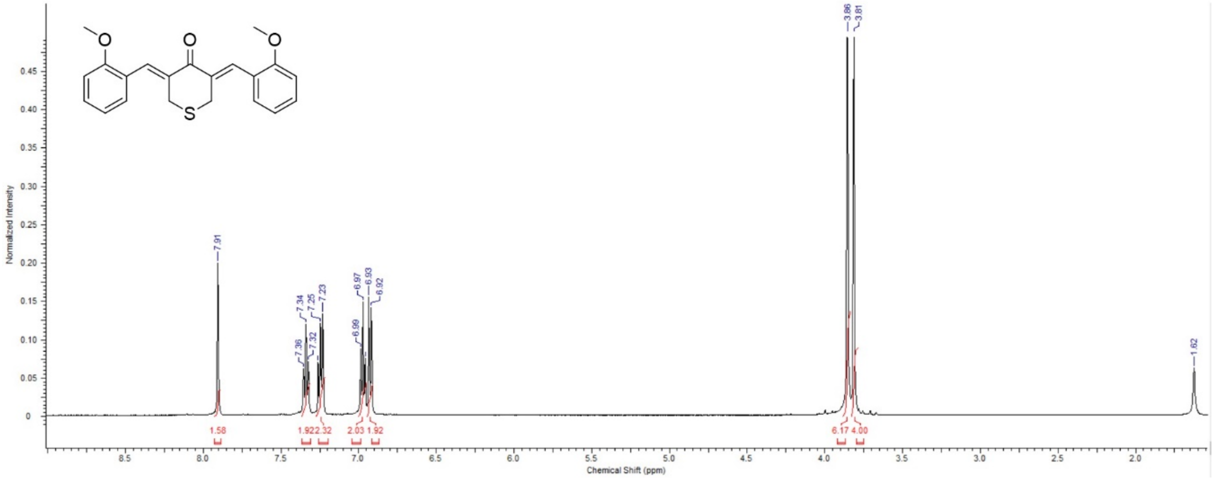

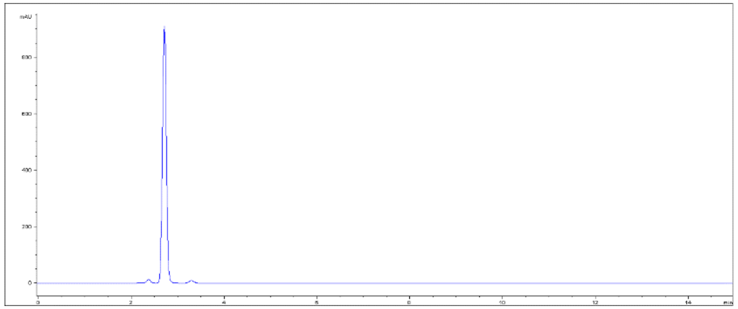

HPLC Conditions:
Column: Thermohypersil C18 Gold (4.6mm ID X 150mm)
Injection volume: 5 µL
flowrate: 1mL/min
Isocratic with 30% water: 70% ACN
Wavelength: 360nm
Retention Time: 2.75 minutes

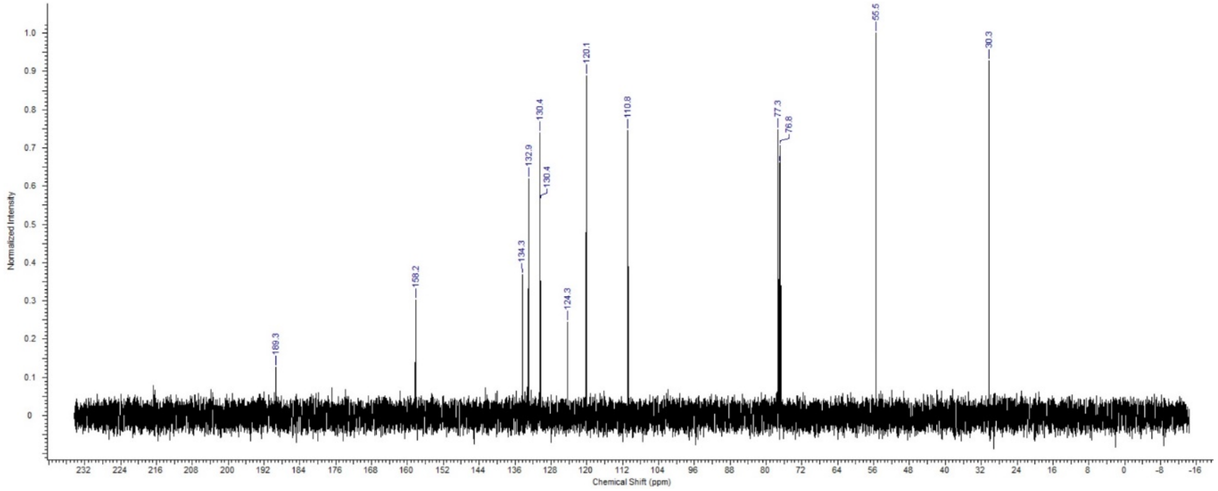

Compound 2e

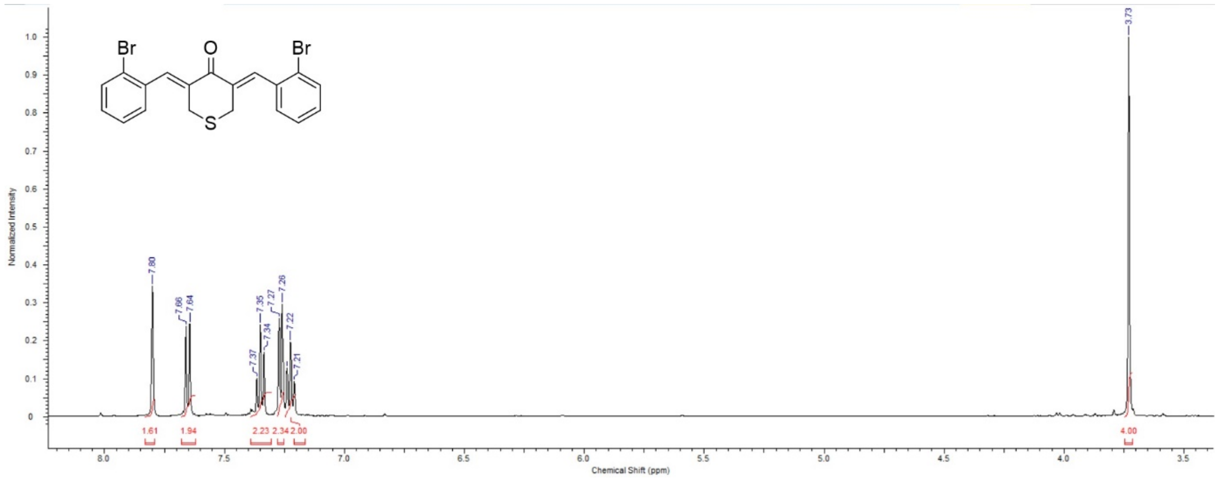

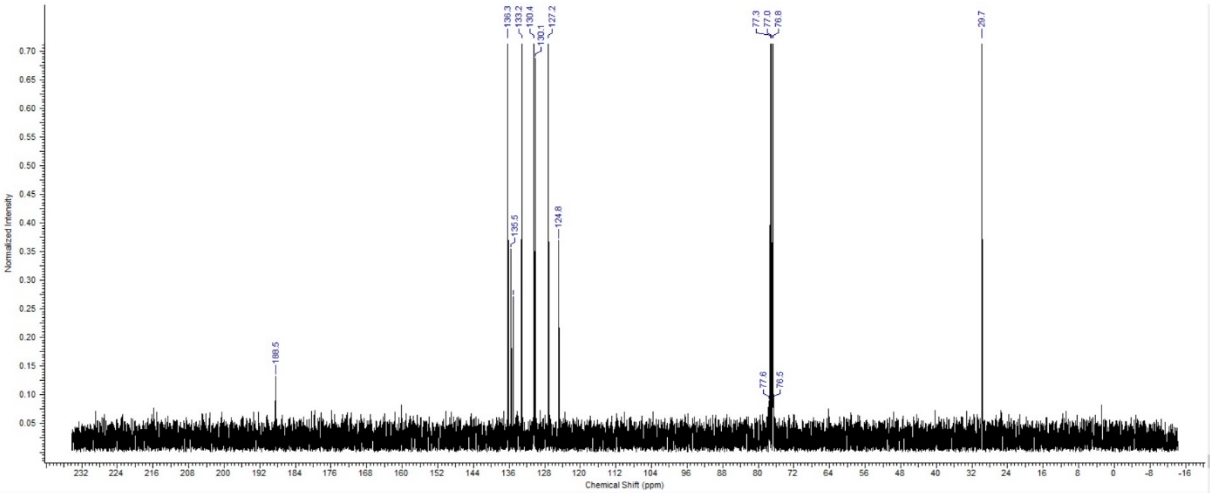

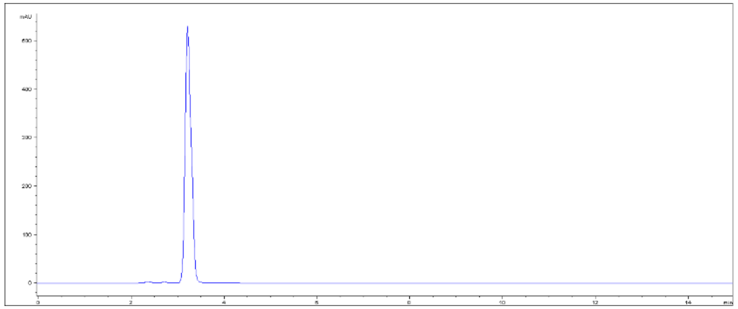

HPLC Conditions:
Column: Thermohypersil C18 Gold (4.6mm ID X 150mm)
Injection volume: 5 µL
flowrate: 1mL/min
Isocratic with 30% water: 70% ACN
Wavelength: 360nm
Retention Time: 3.25 minutes

Compound 2f

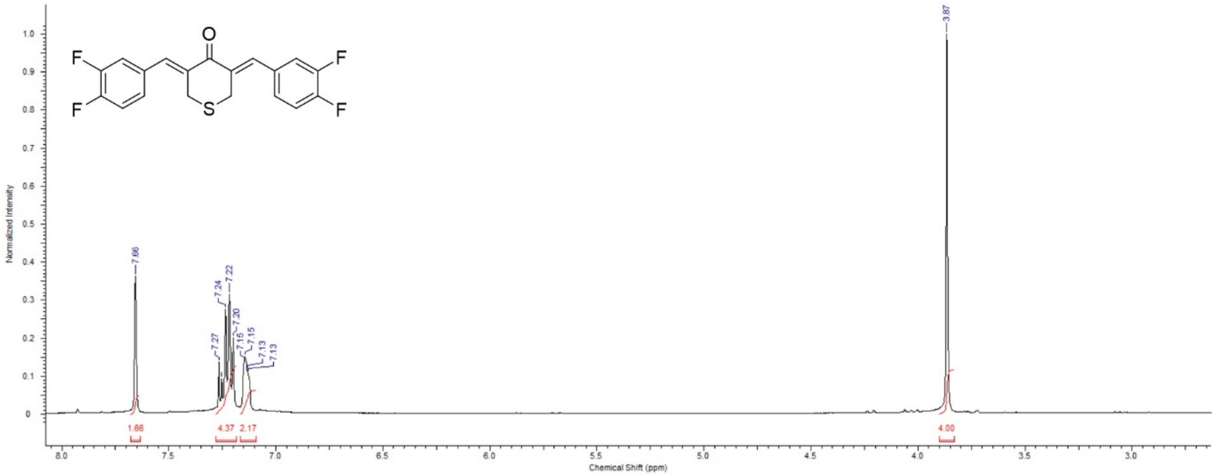

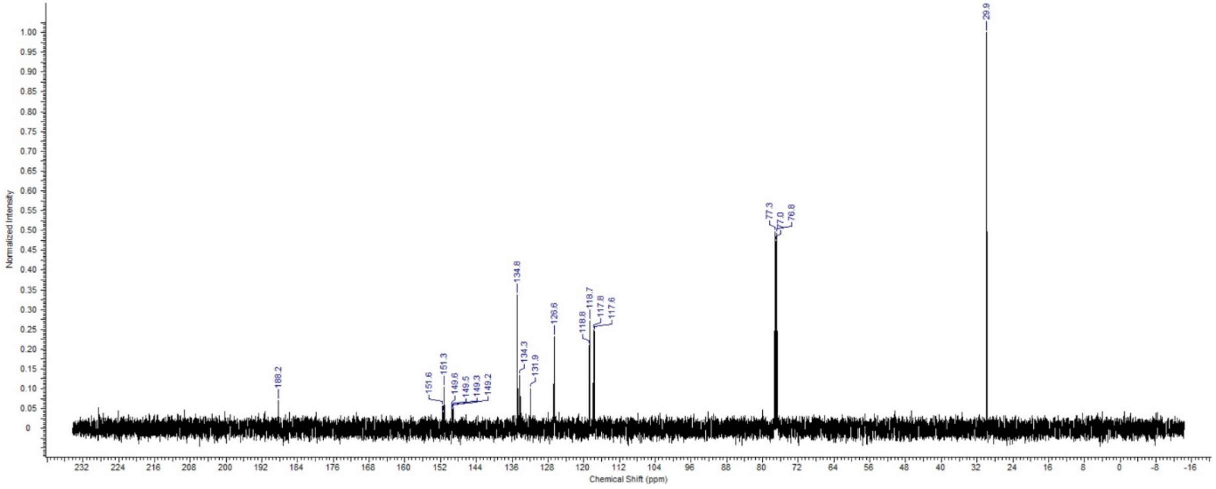

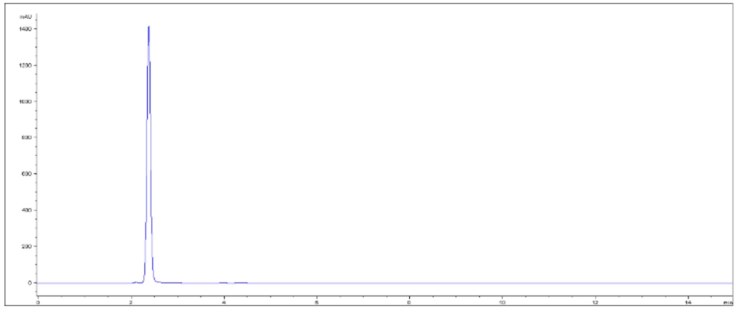

HPLC Conditions:
Column: Thermohypersil C18 Gold (4.6mm ID X 150mm)
Injection volume: 5 µL
flowrate: 1mL/min
Isocratic with 30% water: 70% ACN
Wavelength: 360nm
Retention Time: 2.5 minutes

Compound	X	R	IC_{50} (μM)																Chemo-therapeutic index
			KRAS						BRAF								NHDF		
			T84		SW620		LoVo		HT29		NCI-H508		RKO		LS411N				
			Mean	SD	Mean	SD	Mean	SD	Mean	SD	Mean	SD	Mean	SD	Mean	SD	Mean	SD	
**5-FU**	**-**	**-**	**>10**	**NA**	**>10**	**NA**	**3.3**	**0.91**	**>10**	**NA**	**8.6**	**1.56**	**2.9**	**0.85**	**4.6**	**1.05**	**>20**	**NA**	-
**2a**	C	2-OMe	>10	NA	>10	NA	>10	NA	>10	NA	>10	NA	>10	NA	>10	NA	>20	NA	-
**2b**	C	2-Br	>10	NA	8.9	1.31	>10	NA	>10	NA	7.8	1.14	>10	NA	>10	NA	>20	NA	-
**2c**	C	3,4-F	3.3	1.07	3.2	1.11	3.3	0.67	5.4	0.45	3.3	0.87	2.6	0.96	3.7	0.7	12.9	2.32	2.3-5.0
**2d**	S	2-OMe	>10	NA	>10	NA	6.9	0.66	>10	NA	5.3	0.81	7.1	1.34	5.8	0.7	9.7	1.66	1.4-1.8
**2e**	S	2-Br	1.6	0.36	1.4	0.3	1	0.17	2.1	0.38	1.1	0.36	1.2	0.15	1.3	0.36	7.1	0.61	3.4-7.1
**2f**	S	3,4-F	>10	NA	2.9	0.82	5.1	0.91	>10	NA	6.2	1.23	2.4	0.67	5.9	0.98	2	0.99	0.3-0.8

NA = not available
